# Separable Roles for Neur and Ubiquitin in Delta Signalling in the *Drosophila* CNS Lineages

**DOI:** 10.3390/cells12242833

**Published:** 2023-12-14

**Authors:** Konstantina Kalodimou, Margarita Stapountzi, Nicole Vüllings, Ekaterina Seib, Thomas Klein, Christos Delidakis

**Affiliations:** 1 Department of Biology, University of Crete, 700 13 Heraklion, Greece; nantia_kal@live.com; 2 Institute of Molecular Biology and Biotechnology, Foundation for Research and Technology-Hellas, 700 13 Heraklion, Greece; mas@imbb.forth.gr; 3 Institute of Genetics, Heinrich-Heine-Universitaet Duesseldorf, 40225 Duesseldorf, Germany

**Keywords:** Delta, Neuralized, Hey, Notch, ubiquitylation, endocytosis, asymmetric cell division, *Drosophila*, neurodevelopment

## Abstract

The execution of a Notch signal at the plasma membrane relies on the mechanical force exerted onto Notch by its ligand. It has been appreciated that the DSL ligands need to collaborate with a ubiquitin (Ub) ligase, either Neuralized or Mindbomb1, in order to exert this pulling force, but the role of ubiquitylation per se is uncertain. Regarding the Delta–Neur pair, it is documented that neither the Neur catalytic domain nor the Delta intracellular lysines (putative Ub acceptors) are needed for activity. Here, we present a dissection of the Delta activity using the Delta–Notch-dependent expression of Hey in newborn *Drosophila* neurons as a sensitive in vivo assay. We show that the Delta–Neur interaction per se, rather than ubiquitylation, is needed for activity, pointing to the existence of a Delta–Neur signaling complex. The Neur catalytic domain, although not strictly needed, greatly improves Delta–Neur complex functionality when the Delta lysines are mutated, suggesting that the ubiquitylation of some component of the complex, other than Delta, can enhance signaling. Since Hey expression is sensitive to the perturbation of endocytosis, we propose that the Delta–Neur complex triggers a force-generating endocytosis event that activates Notch in the adjacent cell.

## 1. Introduction

Notch is a fundamental signaling pathway used in multiple contexts of cell fate decisions across the metazoan phyla [1,2]. Notch ligands or DSL proteins (Delta Serrate Lag-2) are tethered to the plasma membrane and need to engage in trans (from adjacent cells) with the Notch receptor in order to activate Notch signal transduction. This is achieved by a series of proteolytic cleavages of the receptor that result in the release and delivery of the Notch intracellular domain (Nicd) to the nucleus to activate the expression of its target genes [3]. The Notch molecule is presented at the plasma membrane in an “off” state, with the site used for the tightly regulated extracellular S2 cleavage buried in its negative regulatory region (NRR) domain [4,5,6]. It has been shown that the mechanical force applied to the Notch extracellular domain (Necd) causes a conformational change to the NRR, which exposes the S2 cleavage site to a constitutively active ADAM metalloprotease [7].

How DSL proteins generate the pulling force needed to activate Notch is a topic of intense study. It has long been appreciated that the DSLs physically associate with ubiquitin ligases, either Mindbomb1 or Neuralized, and this triggers DSL endocytosis [8,9,10,11,12,13]. Ubiquitin, either as a mono-adduct or as a poly-Ub chain, is known to act as an endocytic signal at the plasma membrane, as well as a sorting signal in endosomal compartments (reviewed in [14,15]). The endocytosis of DSL proteins is thought to provide the necessary force to deform the trans-bound Notch NRR [16]. Although the ubiquitylation of the intracellular domains (icds) of Delta and Ser by both Neur and Mib1 has been demonstrated [9,17,18,19], its role in ligand activation is not clear [20,21].

*Drosophila* affords a plethora of sophisticated tools to study these mechanisms. These have been used to dissect the contributions of core pathway components and endocytic factors in the execution of the DSL–Notch signal. In a minimalist approach, Langridge et al. [22] replaced the binding domains of Delta and Notch with other interacting moieties (keeping only the icds and the Notch NRR intact) and evaluated the signaling by these synthetic ligand–receptor pairs in the wing epithelium. They showed that productive signaling depends on the ability of the ligand to enter an Epsin-dependent endocytic pathway. This exerts force on the receptor, as replacing the NRR with another force-sensitive domain modulates the sensitivity of the synthetic Notch receptor to its ligands [22]. This elegantly shows that the need for a force is an intrinsic characteristic of the NRR and does not depend on the specifics of the ligand–receptor binding.

Crucially, the Langridge et al. study shows that compromising Delta’s endocytic activity abolishes its ability to exert a force and produce a signal. However, the specific requirements for signaling in the *Drosophila* wing disk cannot necessarily be extrapolated to other systems. Different synthetic ligand–Notch pairs tested in mammalian cell culture did not recapitulate the necessity of ligand endocytosis for signal generation [21,23]. Meanwhile, studies of developmental processes in different *Drosophila* tissues have provided clues on the context dependence of Notch signal emission. Indeed, Clathrin and Auxilin are needed in the Delta-sending cell in wing and eye epithelia, but are dispensable in egg chambers [24,25]. We recently showed [20] that the disruption of Delta ubiquitylation (by replacing all icd lysines with arginines) strongly reduces the signaling ability of Delta in the wing but does not affect it in the CNS neuroblast (NB) lineages or the selection of PNS precursors. Since the latter two processes differ from the wing in the fact that they are Neur- rather than Mib1-dependent [10,11,12,26,27,28], we speculate that the two E3 ligases function via different mechanisms, with Neur being less reliant on ubiquitylation than Mib1. Here, we test this hypothesis by extending our gamut of Delta and Neur variants, which are examined in the context of CNS lineages. We show that Delta–Neur interaction per se is more important than ubiquitylation, which is dispensable for a basal level of signaling. We further show that ubiquitin on moieties other than the Delta icd is sufficient for strong signal emission, as long as Neur is also present. Together with the fact that dynamin is needed for this process (although Epsin is dispensable), our results lead us to propose that Neur acts as an endocytic adaptor of Delta and that the Delta–Neur complex is more active when modified by Ub moieties, either on the Delta icd or elsewhere. Finally, we present data showing that a similar mechanism is at play during the lateral inhibition of NB birth in the embryo, another Neur-dependent Notch-mediated process.

## 2. Materials and Methods

### 2.1. Generation of Constructs

Dl-HA was used to generate Dl^i1ala^-HA (NEQNAV to AAAAAA) and DlK2R-HA was used to generate DlK2Ri2ala-HA (IRNTWDR to AAAAAAA) by site-directed mutagenesis using the following primers (Table 1), which annealed to both strands (fwd and rev) of the site to be mutated.

Site-directed mutagenesis was performed using the Pfu thermostable DNA polymerase on a plasmid template and both forward (fwd) and reverse (rev) mutagenic primers in the same synthesis reaction on a thermocycler. The mutated product was then assembled onto a full-length Delta cDNA in the pUAST-attB vector [29] by standard restriction enzyme fragment ligation. The final constructs were sequence-verified and subsequently inserted into the M{3xP3-RFP.attP’}ZH-51C attP landing site (attP-ZH51C for short) [29]. All other constructs are described in Berndt et al. (Dl-HA and DlK2R-HA variants—inserted in attP-ZH51C), Daskalaki et al. (UAS-Dli1/2 and UAS-Dli1, generated by random P-element insertion), Wang and Struhl (Dl-LDL, also P-element insertion), or Pavlopoulos et al. (Neur variants, also P-element insertions) [8,9,20,30].

### 2.2. Drosophila Stocks and Genetics

MiMIC, Gal4, and UAS lines:

w*; P{GAL4-da.G32}UH1, Sb^1^/TM6B, Tb^1^ (RRID:BDSC_55851) [31];

y1 w*; Mi{PT-GFSTF.1}neur^MI01824-GFSTF.1^/TM6C, Sb^1^ Tb^1^ (RRID:BDSC_59781) [32];

y1 w*; Mi{PT-GFSTF.1}Delta^MI04868-GFSTF.1^/TM6C, Sb^1^ Tb^1^ (RRID:BDSC_59819) [32];

y1 w*; Mi{PT-GFSTF.0}Ser^MI05334-GFSTF.0^/TM6C, Sb^1^ Tb^1^ (RRID:BDSC_59824) [32];

UASDl-HA/CyOwglacZ [20];

UASDlK2R-HA/CyOwglacZ [20];

UASDl^i1ala^-HA/CyOwglacZ (this study);

UASDl^i2ala^-HA/CyOwglacZ [20];

UASDlK2R^i1ala^-HA/CyOwglacZ [20];

UASDlK2R^i2ala^-HA/CyOwglacZ (this study);

UAS-Dl-LDL [30];

UAS-Dli1/2-V5 [9];

UAS-Dli1-V5 [9];

UAS-EGFP-neur [12];

UAS-neurΔRING-GFP (FlyBase: P{UAS-neur.DeltaRING::EGFP}) [8];

UASDl-HA UAS-EGFP-neur/CyOwglacZ (recombinant developed in this study);

UASDlK2R-HA UAS-EGFP-neur/CyOwglacZ (recombinant developed in this study);

UASDl-HA UAS-neurΔRING-GFP/CyOwglacZ (recombinant developed in this study);

UASDlK2R-HA UAS-neurΔRING-GFP/CyOwglacZ (recombinant developed in this study);

UASDl^i2ala^-HA UAS-neurΔRING-GFP/CyOwglacZ (recombinant developed in this study);

UASDlK2R^i2ala^-HA UAS-neurΔRING-GFP/CyOwglacZ (recombinant developed in this study);

da.G32Gal4 *Dl^RevF10^*/TM6B ([32], recombinant developed in this study);

da.G32Gal4 *neur^1^*/TM3 ([32], recombinant developed in this study);

da.G32Gal4*neur^1^cu Delta^RevF10^*/TM6C ([32], recombinant developed in this study);

hs-FLP; act-FRT > STOP > FRT-Gal4, UAS-GFP (developed by Magadi et al. [33] from RRID:BDSC_4780);

UASshiDN/TM6B (RRID:BDSC_5822);

*lqf^1227^* FRT2A/TM6B [30].

All mutant alleles of mib1, neur, lqf, Delta, and Ser used in this study are described in FlyBase (http://flybase.org/) (accessed on 16 October 2023).

### 2.3. Crosses for Mosaic Analysis

Mosaic analysis with a repressible cell marker, MARCM, system:

Appropriate crosses were maintained at 25 °C, and clones were induced by subjecting the F1 to 1 h heat shock (37 °C), 24–72 h AEL. CNSs were dissected from late L3 wandering larvae three days post-heat shock.

The following MARCM stocks were used:

Stock 1: y w hs-FLP122 tubPGal4 UAS-GFP-6xnls; FRT82B tubPGal80/TM6B (kindly provided by Gary Struhl, Columbia University);

Stock 2: y w hs-FLP122 tubPGal4 UAS-GFP-6xnls; mib^EY9780^ FRT82B tubPGal80/TM6B (generated by us, starting from stock 1);

Stock 3: y w hs-FLP122 tubPGal4 UAS-GFP-6xnls; tubPGal80 FRT2A/TM6B (kindly provided by Gary Struhl, Columbia University);

FRT82B 2xπMyc/TM6B (RRID: BDSC_1459) was used for marked wt clones (control) by crossing to stock (1).

To generate Dl Ser clones, females from stock 1 were used. Males were w/Y; UAS-Dlvariant/+; FRT82B *Dl ^RevF10^ e Ser^RX106^*/TM6B.

To generate *neur Dl Ser* clones, females from stock (1) were used. Males were either w/Y; UAS-Dlvariant/+; FRT82B *neur^1^ cu Dl^RevF10^ e Ser^RX106^*/TM6B or w/Y; UAS-Dlvariant UAS Neur variant/+; FRT82B *neur^1^ cu Dl^RevF10^ e Ser^RX106^*/TM6B.

To generate *mib1* homozygous mutants with *Dl Ser* clones, females from stock 2 were crossed to males.

yw/Y; UAS-Dlvariant/+; *mib^EY9780^* FRT82B *Dl^RevF10^ e Ser^RX106^*/TM6B.

Larvae that carried the Dl variant were selected by virtue of the 3xP3-dsRed marker, found in the attP-ZH51C landing site, which is expressed strongly in larval cortex glia. For Dl variants not inserted in attP-ZH51C, staining with anti-Dl was used to select CNSs bearing the UAS-Dl transgene.

For *lqf* clones, females from stock 3 were crossed to *lqf^1227^* FRT2A/TM6B males.

Flip-out clones:

We crossed hs-FLP; act-FRT > STOP > FRT-Gal4, UAS-GFP flies with UAS shiDN/TM6B. First- to second-instar larvae were subjected to 30 min heat shock (37 °C), to generate clones.

### 2.4. Crosses for Lateral Inhibition Assay in the Embryonic Neuroectoderm

The following crosses were set in cages 

UAS-DlHA/CyO, wglacZ; FRT82B *Dl^RevF10^ e Ser^RX106^*/MKRS × da.G32Gal4 *Dl^RevF10^*/TM6B;

UAS-DlK2RHA/UAS-DlK2RHA; FRT82B *Dl^RevF10^ e Ser^RX106^*/MKRS × da.G32Gal4 *Dl^RevF10^*/TM6B;

FRT82B *Dl^RevF10^ e Ser^RX106^*/+ × daGal4 *Dl^RevF10^*/TM6B;

UAS-EGFP-neur/UAS-EGFP::neur; FRT82B *neur^1^ cu e*/+ × da.G32Gal4 *neur^1^*/TM3;

UAS-neurΔRING-EGFP/UAS-neurΔRING-EGFP; FRT82B *neur^1^ cu e*/+ × daGal4 *neur^1^*/TM3;

FRT82B *neur^1^ cu e*/TM6B × da.G32Gal4 *neur^1^*/TM3;

UAS-DlHA UAS-EGFP-neur/+; FRT82B *neur^1^ Dl^RevF10^ e Ser^RX106^*/MKRS × daGal4 *neur^1^ cu Dl^RevF10^*/TM6C;

UAS-DlK2RHA UAS-neurΔRING-EGFP/UAS-DlK2RHA UAS-neurΔRING-EGFP; FRT82B *neur^1^ Dl^RevF10^ e Ser^RX106^*/MKRS × da.G32Gal4 *neur^1^ cu Dl^RevF10^*/TM6C;

FRT82B *neur^1^ Dl^RevF10^ e Ser^RX106^*/TM6B × da.G32Gal4 *neur^1^ cu Dl^RevF10^*/TM6C.

We ensured that the only lethal combination of chromosomes in the F1 was the Dl or neur homozygotes, since we could not otherwise genotype the embryonic cuticles.

### 2.5. Immunohistochemistry—Antibodies

Fixation, blocking, and antibody incubation were performed according to standard protocols. We used the following primary antibodies:

gp anti Dpn [33], DSHB Cat# Rat-Elav-7E8A10 anti-elav, RRID:AB_528218, mouse anti-Dl (DSHB Cat# c594.9b, RRID:AB_528194), rabbit anti-GFP (MINOTECH, discontinued), goat anti-GFP: Abcam ab6673 (RRID:AB_305643), guinea pig anti-Hey [28]. Fluorochrome-conjugated secondary antibodies: DyLight™ 405 AffiniPure Donkey Anti-Guinea Pig IgG (H+L) Jackson Immunores. 706-475-148(RRID: AB_2340470), donkey anti-goat Alexa Fluor^®^ 488 Life Biotech (Mol. Probes) A11055 (RRID AB_2534102), goat anti-Rb Alexa Fluor^®^ 488 Life Biotech (Mol. Probes) A11034 (RRID AB_2576217), donkey anti-rat Cy3-AffiniPure F(ab’)2 Frag Jackson Immunores. 712-166-153 (RRID: AB_2340669), goat anti-gp Alexa Fluor^®^ 647 Life Biotech (Mol. Probes) A21450 (RRID AB_2735091), donkey anti-mouse Alexa Fluor^®^ 647 Invitrogen A31571 (RRID AB_162542), donkey anti-gp Alexa Fluor^®^ 647-AffiniPure F(ab’)2 Frag Jackson Immunores. 706-606-148 (RRID: AB_2340477).

### 2.6. Confocal Microscopy—Ganglion Mother Cells’ Asymmetric Cell Division (GMC-ACD) Assay Scoring and Image Analysis

Images were acquired with a Leica TCS SP8 laser scanning confocal microscope (IMBB-FORTH imaging facility). MARCM lineages or flip-out clones from stained CNSs were scanned at 40× (HC PL APO CS2 40×/1.30 OIL) with zoom 1–4×, at a z-step of 0.7–1 μm. Each GFP-marked lineage spanned several optical sections. We characterized each GFP-marked lineage as positive (multiple Hey+ neurons), weakly positive (1 or 2 Hey+ neurons), or negative (only Hey− neurons), by navigating manually through all optical sections of the lineage, with the Leica LAS-X software. We excluded the mushroom body lineages from our scoring, since we had earlier shown [28] that they express Hey without any input from Notch signaling. Images with examples of the mutant lineages presented here are single section images, where scale bars were inserted using ImageJ, and the final assembly, slight contrast enhancement, and clone outlining were performed with Adobe Photoshop. In Figure S3, we show cropped images of 125 × 125 px showing the neuroblast and several GMCs/neurons from one lineage. These images were obtained using the 40× objective lens (HC PL APO CS2 40×/1.30 OIL) with zoom = 3. GMC-ACD statistics: for each genotype, the experiment was repeated 2–3 times and all the lineages scored were pooled together. The result is shown in graph bars as a percentage of each category. We performed pairwise comparisons between genotypes, using the chi-square goodness-of-fit test. Statistical significance: two tailed *p*-value, non-significant differences for *p* > 0.05, (*) 0.01 < *p* < 0.05, (**) 0.001 < *p* < 0.01, (***) 0.0001 < *p* < 0.001, (****) *p* < 0.0001. A summary of all comparisons is shown in Table S1.

### 2.7. Lateral Inhibition Assay in the Embryonic Neuroectoderm

Embryo collection—cuticle preparations: 150–270 embryos per replicate per genotype were transferred into a new agar plate 12 h after egg laying. More than 36 h AEL, we counted the number of hatched and unhatched embryos under the stereoscope. All unhatched embryos were dechorionated on the plate, with 50% bleach. After several rinses with water, the unhatched embryos were mounted in Hoyer’s medium [34] and incubated for at least 4 h at 65 °C. For each genotype, we performed 4–5 replicates (separate embryo transfers) but we included in our analysis the 2–3 replicates that showed minimal embryo loss during the described manipulation.

Phenotypic analysis—scoring and statistics: We observed the cuticle preparations with a 5× lens with the transmitted light Leica inverted microscope and documented the following categories: wild type, weak, intermediate, strong, other, and unfertilized embryos (which were completely transparent and maintained only their vitelline membrane). After subtracting the unfertilized embryos from the total and correcting for any embryo losses during handling, we were able to estimate the percent lethality. We saw that in none of the genotypes was the number of dead embryos less than the expected one quarter of the fertilized, so no rescue to survival was inferred.

## 3. Results

### 3.1. Delta–Neur Signaling in Newborn Neurons Can Function in the Absence of the Delta Lysines and Mib1

**Figure 1:**
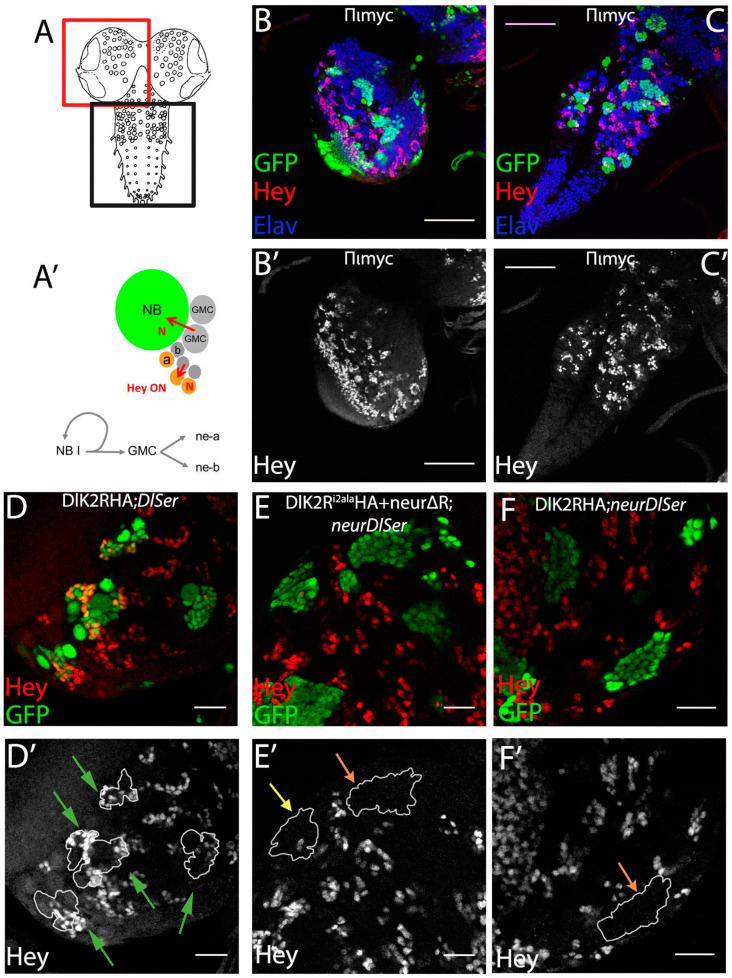
The Ganglion Mother Cells’ Asymmetric Cell Division (GMC ACD) assay (**A**) Graphic of the larval CNS; the red box depicts a brain hemisphere and the black box the ventral nerve cord. (**A’**) Graphic of a neuroblast (NB) lineage: the NB undergoes asymmetric divisions to self-renew and generate ganglion mother cells (GMCs). Each GMC divides asymmetrically to give rise to neuron type a (Hey-positive Elav-positive, Notch-ON) and neuron type b (Hey-negative Elav-positive Notch OFF); red arrows show Notch signaling events. (**B’**,**C’**) Overview of control mosaic CNS (FRT 82B) with no mutations. (**B**) Brain hemisphere. (**C**) Ventral nerve cord. Green: GFP clonal marker; red: Hey, marks only young Notch-ON neurons; Blue: Elav, marks all neurons, but not NBs or GMCs. Scale bar 60 μm. (**D**–**F**) Examples of genotypes that produce strong (**D**), moderate (**E**), or no (**F**) rescue of Hey expression. Single sections from enlarged areas of the central brain containing GFP-marked clones expressing a Dl variant in various mutant backgrounds, as indicated (for a description of Dl variants, see Figure 3). Red: Hey. (**D’**–**F’**) Hey pattern alone; the borders of the clones are drawn in white. Green, yellow, and orange arrows point to positive, weakly positive, and negative lineages respectively; the same color code is used in the bar charts of Figure 4, Figure 5, Figure 6 and Figure 7. Scale bar: 20 μm.

**Figure 2:**
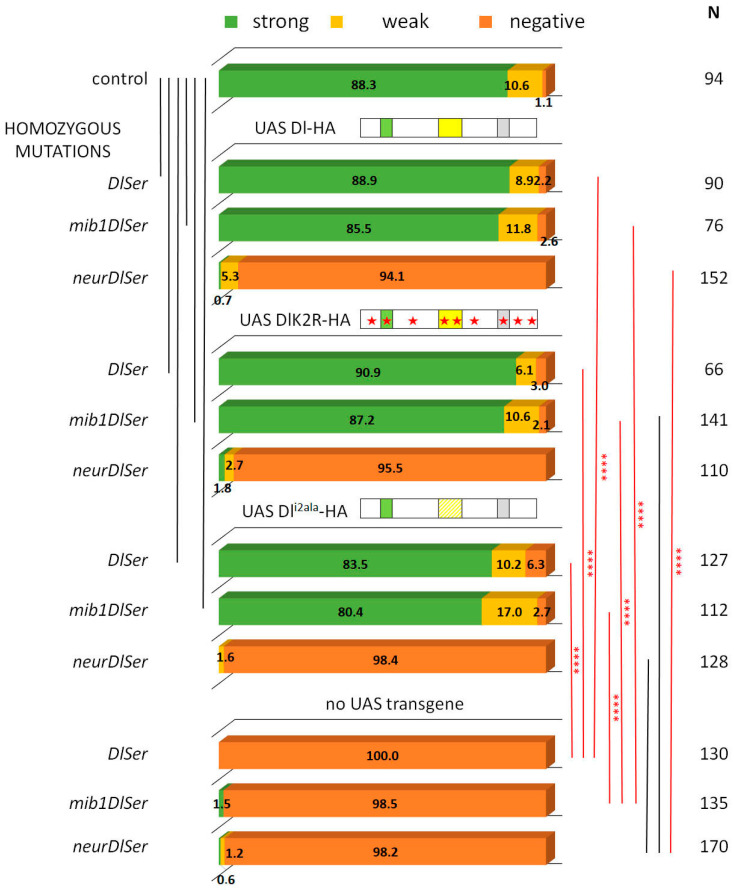
Clonal phenotype classification and quantification for Dl-HA, Dl-K2R-HA, and Dl^i2ala^-HA. Three phenotypic categories are presented as a percentage of the total number (N) of lineages scored: strong (>2 Hey positive cells) is shown in green, weak (1–2 Hey positive cells) is shown in yellow, and negative (no Hey-positive cells) in dark orange. Genotypes (from top to bottom): control (neutral clones); clones expressing Dlwt-HA in *DlSer*, *mibDlSer*, or *neurDlSer* background; clones expressing DlK2R-HA in *DlSer*, *mibDlSer*, or *neurDlSer* background; clones expressing Dl^i2ala^-HA in *DlSer*, *mibDlSer*, or *neurDlSer* background; clones mutant for *DlSer*, *mibDlSer*, or *neurDlSer* without any transgene expression. Selected pairwise comparisons are shown (chi-square): black lines indicate non-significant differences (ns, *p* > 0.05) and red lines significant differences (****): *p* < 0.0001. For more comparisons, see Table S1. Dl transgenes’ cartoons as in Figure 3.

**Figure 3:**
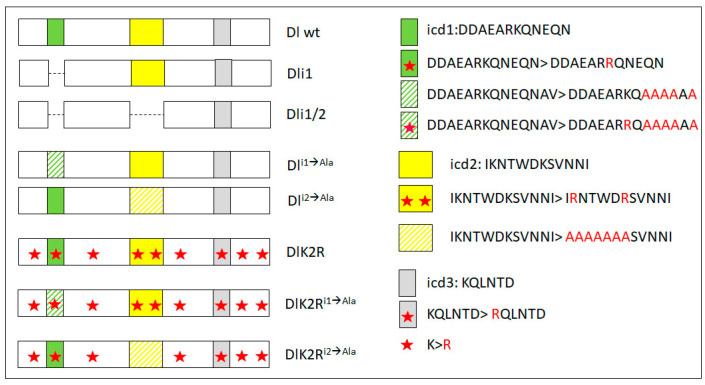
Schematic view of the intracellular domain of all Delta variants used in this study (on the left). Only the intracellular domain is shown, as the extracellular and transmembrane domains are intact in all variants used in this study. icd1 stands for intracellular domain 1 and corresponds to residues 630–641, icd2 to residues 682–793, icd3 to residues 742–747. icd1–3 are three strongly conserved motifs across insects [9]. Dli1 and Dli1/2 are precise deletions of icd1 or both icd1 and icd2, respectively. On the right, the amino acid changes present in the remaining variants are shown, highlighted in red. Hatched icd boxes denote changes in part of the motif to alanines, as shown on the right. Red stars denote changes of lysines to arginines. In addition to the nine lysines shown here, there are three more lysine residues directly following the transmembrane domain (at the N-terminus of the segment of Delta shown here—the putative stop-transfer sequence); these, too, have been converted to arginines in all variants bearing the K2R label.

The process of asymmetric fate adoption by the siblings born after a Ganglion Mother Cell (GMC) division in the *Drosophila* CNS relies mostly on Neur and Delta [28], both of which are prominently expressed in all NB lineages (the stem cell and its immature progeny; Figure 1A,A’ and Figure S1). In contrast, the other Notch ligand, Ser, is primarily expressed in glial cell populations, although it is also detected in a subset of immature neuron lineages. We genetically ablated both Delta and Ser and simultaneously expressed a Delta variant using the MARCM technique, in order to assess the variant’s efficacy in generating a signal, when it is the only source of Notch ligand. In order to be able to discern fine differences, most UAS-Dl variants were inserted in the same attP site (attP-ZH51C, see Methods for the few exceptions) and we scored at least 100 MARCM clones per genotype. We classified clones into three categories, exemplified in Figure 1: non-expressing (no Hey-positive cells, orange arrows in Figure 1E’,F’), weak (1–2 Hey-positive cells, yellow arrow in Figure 1E’), or strong (>2 Hey-positive cells, green arrows in Figure 1D’). In a normal late larval CNS (Figure 1B,C), 88% of 3-day-old control clones (GFP-marked descendants of a single stem cell, with no further genetic manipulation) fall within the strong category, whereas 11% are scored as weak (Figure 2). In contrast, *Dl Ser* mutant clones are devoid of Hey expression. Using this assay, we confirmed our earlier result that both UAS-Dl-HA (a wt HA-tagged Delta transgene) and UAS-DlK2R-HA (a Delta variant with all 12 intracellular lysines mutated into arginines) are capable of supplying full levels of ligand activity [20]; the distribution of clones among the three classes (strong/weak/negative) was indistinguishable from that of wt control clones (Figure 2, Table S1). The entire set of Delta variants used in this study is listed in Figure 3; see the legend for a description of the conserved motifs (icd1, 2, and 3) and the mutations included in each variant.

From our earlier data [28], we know that Neur is the major E3 ligase needed for GMC sibling specification, with Mib1 playing a very minor role. We tested the wt and K2R Dl transgenes in the absence of either *mib1* or *neur*, by recombining strong hypomorphic alleles of these genes in the Dl Ser background. Both Dl-HA and DlK2R-HA showed indistinguishable high activity in the absence of *mib1* (Figure 2), showing that Mib1 is dispensable for Delta signaling in this context. In contrast, both the wt and K2R Dl variants lost their activity in a *neur Dl Ser* background. It therefore seems that the Neur–Dl combination is what provides a sufficient signal in this cellular context, even in the absence of Delta’s intracellular lysines. We had earlier mapped the docking sites for Neur and Mib1 to separate regions within the Dl icd [9], which we call icd1 (or the NxxN motif [35,36]) for Neur and icd2 (or the N-box [18]) for Mib1 (see Figure 3). The dispensability of Mib1 in activating Dl was further validated by the study of the mutant Dl^i2ala^. In this mutant, the icd2 motif on Delta’s intracellular tail is mutated to alanines (IKNTWDK > AAAAAAA). We observed that this mutant behaves exactly like the wt Dl, losing functionality only in the absence of neur (Figure 2).

### 3.2. A Role for Delta Lysines and Mib1 Is Revealed When Delta–Neur Interaction Is Compromised

**Figure 4:**
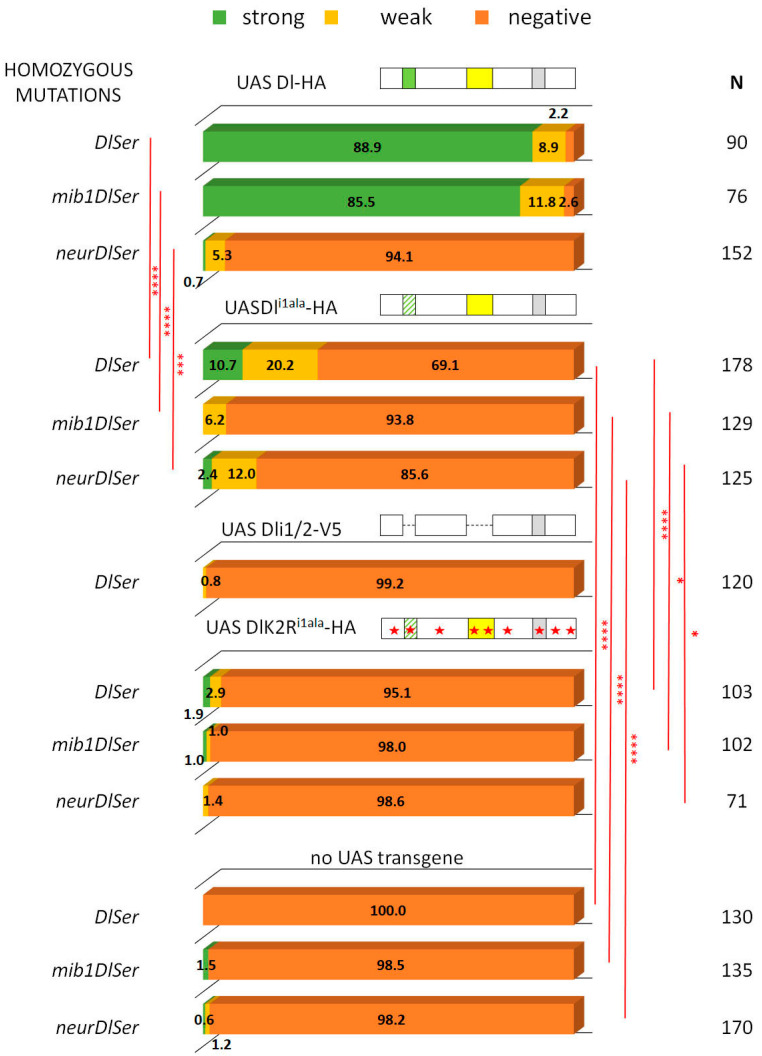
Behavior of Dl^i1ala^ variants in GMC asymmetric cell divisions. Graph bar for the Hey phenotype quantification classified in three categories as in Figure 2. Genotypes (from top to bottom): Dl-HA expression in *DlSer*, *mibDlSer*, or *neurDlSer* clones (same data as Figure 2 for comparison); Dl^i1ala^-HA expression in *DlSer*, *mibDlSer*, or *neurDlSer* clones; Dli1/2-V5 expression in *DlSer* clones; DlK2R^i1ala^-HA expression in *DlSer*, *mibDlSer*, or *neurDlSer* clones; mutant clones for *DlSer*, *mibDlSer*, or *neurDlSer* (same data as Figure 2 for comparison). Selected pairwise comparisons are shown (chi-square): red lines for significant differences (*): 0.01 < *p* < 0.05, (***): 0.0001 < *p* < 0.001, (****): *p* < 0.0001. For more comparisons, see Table S1. Dl transgenes’ cartoons as in Figure 3.

Since Neuralized is indispensable in GMC sibling specification, we would expect that taking out icd1 (Neur’s docking site on Dl’s intracellular tail) would abolish N signaling entirely. To our surprise, we observed that the Dl^i1ala^ mutant retained significant functionality, with 30% of the lineages activating Hey (weak and strong) (Figure 4). This activity could either be due to the residual interaction of the mutant Dl with Neur or due to the activity of Mib1, which had been somehow masked in the experiments with the intact Dl intracellular region. To address these two possibilities, we tested the activity of Dl^i1ala^ in the absence of each of the Ub ligases. Both scenarios were supported by the results, as removing either Mib1 or Neur strongly decreased the signaling capacity of Dl^i1ala^, to low but still detectable levels (6% or 14% of clones are Hey-positive). The ability of Dl^i1ala^ to signal (weakly—14% positive clones) in the absence of neur can be accounted for by a contribution of Mib1, whose docking site (icd2) is intact in this variant. The (even weaker—6% positive clones) ability of Dl^i1ala^ to signal in the absence of mib1, on the other hand, is quite unexpected and suggests that Neur may have a secondary weaker docking site on Delta. Since the Dl^i1ala^ variant has only the NxxN motif mutated and retains the preceding eight amino acids (DDAEARKQ), five of which are highly conserved among the insects, we considered the possibility that Dl^i1ala^ may still weakly interact with Neur. We therefore tested a deletion of the entire icd1 (DDAEARKQNEQN) in the same three mutant backgrounds (*Dl Ser*, *neur Dl Ser*, and *mib1 Dl Ser*). This variant, Dli1 [9], behaved identically to Dl^i1ala^, displaying Mib1-dependent activity, but also retaining a very low level of activity in the absence of mib1 (Figure S2). We therefore propose that the secondary docking site of Neur on Delta is not the residual icd1, but rather the icd2, as the double deletion of both icd1 and icd2 (Dli1/2-V5) renders Delta completely inactive (Figure 4 and [20]).

**Figure 5:**
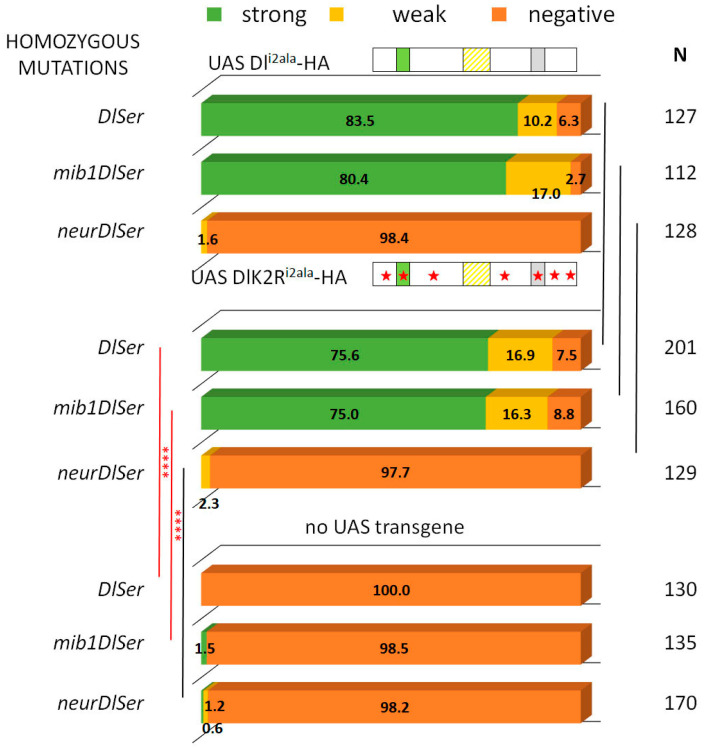
Dl^i2ala^ and DlK2R^i2ala^ variants rescue Hey expression similarly. Graph bar for the Hey phenotype quantification classified in three categories as in Figure 2. Genotypes (from top to bottom): Dl^i2ala^-HA expression in *DlSer*, *mibDlSer*, or *neurDlSer* clones (same data as Figure 2 for comparison); DlK2R^i2ala^-HA expression in *DlSer*, *mibDlSer*, or *neurDlSer* clones; mutant clones for *DlSer*, *mibDlSer*, or *neurDlSer* with no transgene expression (same data as Figure 2 for comparison). Selected pairwise comparisons are shown (chi-square): black lines are used for non-significant differences (ns): *p* > 0.05 and red lines for significant differences, (****): *p* < 0.0001. For more comparisons, see Table S1. Dl transgenes’ cartoons as in Figure 3.

Upon substitution of all intracellular lysines with arginines (K2R version), Dl^i1ala^ lost its activity (DlK2R^i1ala^HA, Figure 4), unlike the lysine-containing Dl^i1ala^HA. Therefore, the Mib1-dependent activation of Dl seems to require the Dl icd lysines and the same seems to hold true for the activation engendered by the weak secondary interaction of Neur with Dl. On the other hand, when the Dl–Neur strong interaction (via icd1) is intact, Dl lysines are dispensable. Besides the DlHA vs. DlK2RHA comparison (Figure 2), this conclusion is also supported by the Dl^i2ala^ vs. DlK2R^i2ala^ pair (absence of icd2, but intact icd1, strong Neur docking motif): their activity was indistinguishable from each other in all mutant backgrounds tested, being high in the presence of Neur and undetectable in the absence of Neur (Figure 5).

To summarize, we observe that both the icd2 and the Delta icd lysines (which were heretofore thought to be dispensable) contribute to Delta activity and this becomes apparent when the Delta–Neur strong interaction is compromised.

### 3.3. The Neur RING Domain Is Important but Not Indispensable for Delta Signaling

We had previously reported that the deletion of the catalytic domain of Neur (neurΔRING) does not affect its activity in GMC progeny signaling [20]. We tested a number of Delta variants co-expressed with UAS-neurΔRING in a neur Dl Ser background to gain more insights into the apparent redundancy of this highly conserved ubiquitin ligase domain.

**Figure 6:**
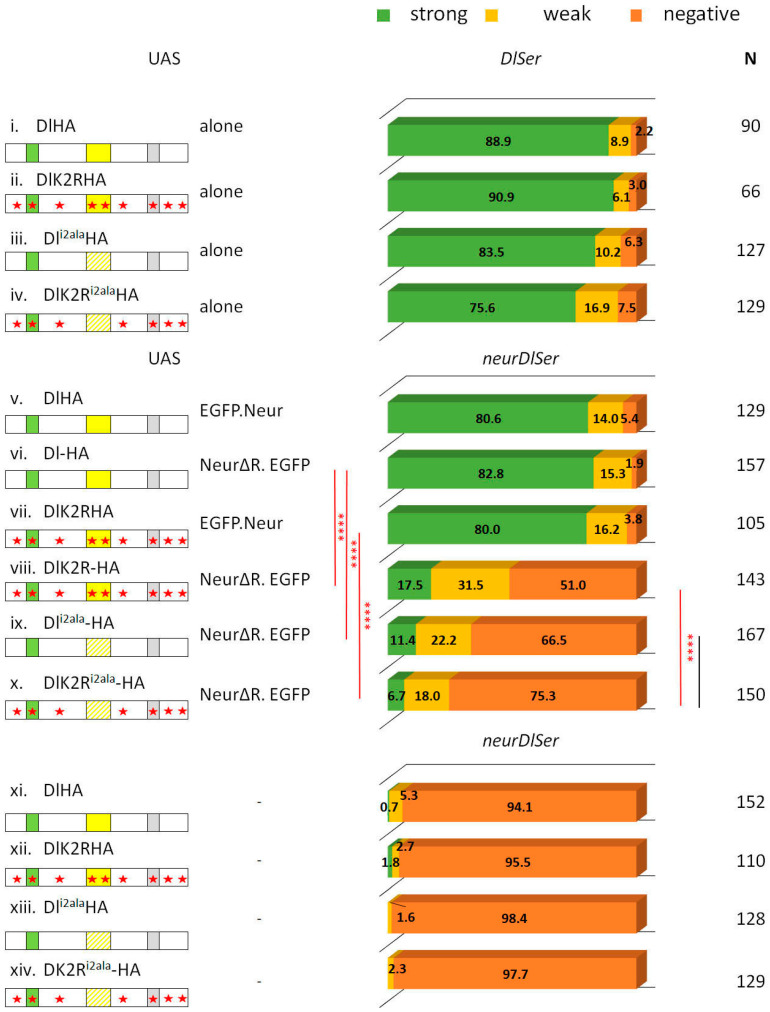
Combination of Delta variants with NeurΔRING. Bar chart for the Hey phenotype quantification classified into three categories as in Figure 2. Genotypes (from top to bottom): *DlSer* clones expressing Dl-HA, DlK2R-HA, Dl^i2ala^, DlK2R^i2ala^ (data from Figure 2 and Figure 5 for comparison); *neurDlSer* clones expressing Dl-HA + EGFP-Neur, Dl-HA + NeurΔRING-EGFP, DlK2R-HA + EGFP-Neur, DlK2RHA + NeurΔRING-EGFP, Dl^i2ala^-HA + NeurΔRING-EGFP, DlK2R^i2ala^-HA + NeurΔRING-EGFP, Dl-HA alone, DlK2R-HA alone, Dl^i2ala^-HA alone, DlK2R^i2ala^-HA alone (last four bars from Figure 2 and Figure 5 for comparison). Selected pairwise comparisons are shown (chi-square): black lines are used for non-significant differences (ns): *p* > 0.05 and red lines for significant differences (****): *p* < 0.0001. For more comparisons, see Table S1. Dl transgenes’ cartoons as in Figure 3.

We observed that NeurΔRING can sustain the full activity of DlHA, thus confirming that the catalytic domain of Neur is dispensable (Figure 6). Since DlHA contains lysines and the docking site for Mib1, we decided to test NeurΔRING on DlK2R^i2ala^. In this background, ubiquitylation should be minimal or even abolished, as there are no Delta lysines (K2R), no ability to recruit the alternative E3 ligase, Mib1 (i2ala), and no catalytic domain on Neur (ΔRING). Although signaling was diminished, 25% of the lineages were still able to activate the Notch target Hey. We conclude that ubiquitylation is not absolutely needed for Delta signaling; what is important is the presence of Neur per se, since only 2% of Hey-positive lineages were obtained with the same Delta variant when neurΔRING was omitted. A plausible scenario is that Neur acts as an adaptor of Delta to the pulling machinery that gives rise to its signaling activity.

The signaling activity of DlK2R^i2ala^+NeurΔRING was not significantly improved upon restoring the Delta lysines (comparing DlK2R^i2ala^ with Dl^i2ala^). These two Delta variants also gave comparable activity to each other in a neur+ background (restoring the Neur catalytic domain; Figure 5). However, in this case, significantly more activity was observed (93–94% Hey-positive lineages) than what we had obtained with the NeurΔRING variant (25–33% Hey-positives). It is, therefore, possible that Neur and Delta interact to generate a pulling complex and Neur ubiquitylates some other substrate, besides Delta, within this complex, in order to strengthen its signaling activity.

If the ubiquitylation of some component of the Neur–Delta complex is needed for full activity, could the apparent dispensability of the Neur catalytic domain on wt Delta be due to the ability of the Delta lysines to be ubiquitylated by alternative machinery, like Mib1? Indeed, the DlHA+NeurΔRING pair gave wt activity, whereas DlK2R+NeurΔRING was weaker (48% Hey-positive vs. 98%; Figure 6). Since the presence of the Delta lysines made no difference in the background of Dl^i2ala^, we hypothesize that Mib1 (in the presence of its icd2 docking site, i.e., in the DlHA and DlK2R pair) can make use of the Delta lysines to strengthen the Delta–NeurΔRING pulling complex via ubiquitylation.

Finally, it is worth pointing out that DlK2R+NeurΔRING shows a significant improvement over the DlK2R^i2ala^+NeurΔRING complex (48% vs. 27% Hey-positive lineages, Figure 6). Why would the presence of icd2 make a difference if its interactor (Mib1) has no Dl lysines to modify? This may be due to the role of icd2 as a secondary Neur docking site. It is possible that the catalytically disabled Dl–Neur complex is more active when Neur contacts both its strong and weak docking sites on Dl. Alternatively, the improved activity of the DlK2R+NeurΔRING pair may be due to Mib1 contributing Ub moieties on substrates other than Dl to strengthen the activity of the DlK2R+NeurΔRING pulling complex. We cannot distinguish between these two scenarios based on our data.

### 3.4. The Role of Endocytosis in Delta Activation in CNS Lineages

Several lines of evidence to date suggest that the pulling force exerted by Delta to activate Notch is generated via endocytosis [24]. The results presented here have shown that ubiquitylation is dispensable for moderate Delta activation, although it does contribute to high levels of activity. Since the ubiquitylation of membrane proteins correlates with their endocytosis [14,15], the diminished need for ubiquitylation in the CNS could reflect that endocytosis is not needed in this tissue, and instead other uncharacterized mechanisms are in place to generate the pulling force needed to activate Notch. We set out to interrogate this possibility by genetically manipulating Delta endocytosis and assaying the effects on Hey expression in CNS lineages.

**Figure 7:**
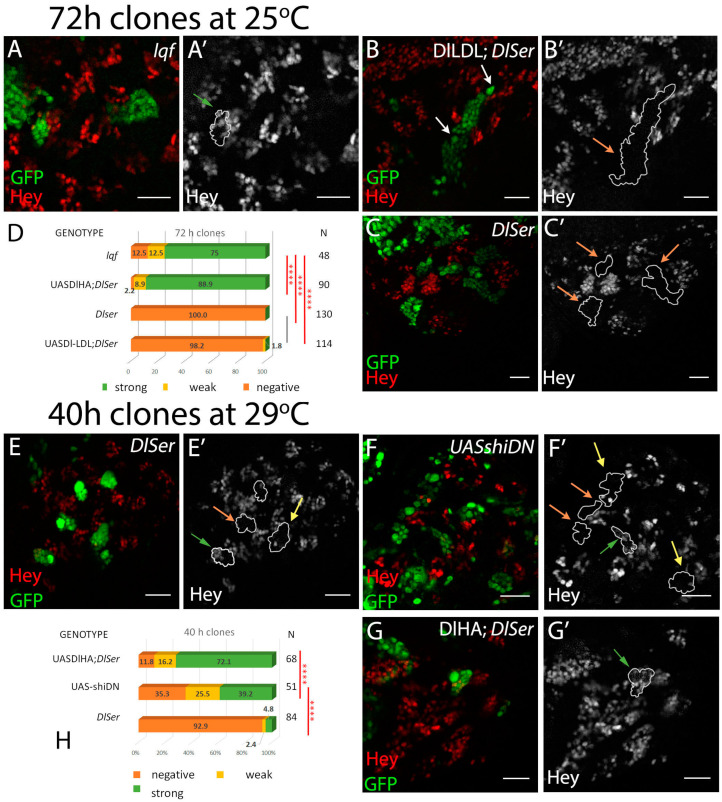
The role of epsin, dynamin, and DlLDL. (**A**–**C**) Single sections of enlarged areas of the central brain showing characteristic examples of 3-day mutant clones for epsin (*lqf*) (**A**) or *DlSer* expressing DlLDL (**B**). Green: GFP, red: Hey. (**A’**) One Hey-positive lineage is highlighted. (**B’**) Two neighboring lineages are shown, with their NB highlighted by white arrows, both of which are Hey-negative. (**C’**) *Dl Ser* control lineages, all negative. (**D**) Graph bar for the Hey phenotype quantification for the genotypes shown in (**A**,**B**). The results from equivalently aged clones from *Dl Ser* or *Dl Ser* + UAS-Dl-HA (from Figure 2) are included for comparison. (**E**–**G**) 40 h clones of the indicated genotypes; animals were kept at 29 °C after clone induction. Green: GFP, red: Hey. (**E’**–**G’**) Hey pattern alone; the borders of the lineages are drawn in white. Green, yellow, and orange arrows point to positive, weakly positive, and negative lineages, respectively. (**E**) *DlSer* mutation abolishes Hey expression 40 h after clone induction, with few escapers. (**F**) Flip-out clones overexpressing a dominant negative allele for *shi*. (**G**) DlHA; *DlSer* restores Hey expression 40 h after clone induction. (**H**) Graph bar for the Hey phenotype quantification for the genotypes shown in (**D**–**F**). Scale bar: 20 μm. In D and H, selected pairwise comparisons are shown (chi-square): black lines are used for non-significant differences: *p* > 0.05 and red lines for significant differences (****): *p* < 0.0001. See also Table S1.

In the wing and eye disks, the connection between endocytosis and Delta activity has been supported by, among others, the inactivity of Delta in Epsin mutant clones [30,37]. Epsin, encoded by lqf in *Drosophila*, is an endocytic adaptor protein that is able to recognize Ub on plasma membrane cargoes and target them for endocytosis. We generated *lqf* clones and saw a minor defect in the expression of Hey in CNS lineages (88% of Hey-positive lineages) (Figure 7A). This contrasts the severe defects of similar clones in wing DV boundary, sensory organ precursor, and eye photoreceptor determination, all Notch-mediated processes. This may indicate an endocytosis-independent function of Delta in neuronal precursors; alternatively, since Epsin is one of several Ub adaptors encoded in the *Drosophila* genome, it may simply indicate that Delta endocytosis uses a different endocytic adaptor in these cells. Several instances of Ub-interacting domains, like UIM, UEV, or UBA [15], are encountered in the proteome, both in endocytic adaptors, like Eps15, Hrs, Stam, or Tsg101, and in less well-characterized proteins.

Another way to manipulate Delta endocytosis takes advantage of the fusion of heterologous endocytic motifs to the extracellular and transmembrane domains of Delta. One such fusion used the mammalian LDL receptor endocytic motif, a Ub-independent constitutive signal, for rapid endocytosis and recycling. Dl-LDL, as this synthetic ligand was named, was able to induce a wing DV boundary in the absence of Epsin [30]. When we tested Dl-LDL in the GMC asymmetric cell division assay, we recovered only 2% of weakly positive lineages (Figure 7D). This means that endocytosis is not sufficient to generate a signal in this context and confirms that the interaction with Neur (absent in Dl-LDL) is of paramount importance. We had earlier obtained the same result (inactivity of Dl-LDL) in another Neur-mediated instance of Notch signaling, the lateral inhibition of sensory organ precursors in the wing disk [9].

A third approach was aimed at blocking endocytosis at an early step, by compromising the abscission of the vesicle from the plasma membrane. The GTPase dynamin has been shown to be crucial in this process. We clonally expressed UAS-shiDN, a dynamin variant locked in its inactive GDP-bound form. Unfortunately, we were not able to recover viable animals after 3 days of clonal shiDN expression, due to the multitude of processes affected by dynamin’s function. By testing CNSs earlier, 40 h after clone induction, when larvae were still alive, we noticed a significant reduction in Hey-positive lineages (65%) compared to equally aged clones expressing wt Delta in a Dl Ser background (88% positive; Figure 7H). These results are consistent with the endocytosis of Delta being needed in this instance of signaling, similar to all others tested. Taken together with the Dl-LDL and lqf results, they point to a special pathway of dynamin-dependent endocytosis that requires the presence of Neur as both an adaptor and an enzyme, but largely dispenses with Epsin, as the mechanism via which Delta emits its signal in this setting.

We surveyed the subcellular localization of four Delta variants in CNS lineages mutant for endogenous neur, Dl, and Ser. As the tubulin promoter (aTub84B) used in the MARCM system for mosaic clones’ generation seems to vary quite extensively in expression strength among different CNS lineages, we present a collage of 10 random clones per variant (Figure S3). The four variants, DlHA, Dl^i2ala^, DlK2R, and DlK2R^i2ala^, were tested either singly or in the presence of NeurΔRING. Internalization was quite variable from lineage to lineage within the same sample, making it difficult to draw solid conclusions. Nonetheless, by inspecting a large number of lineages, we can discern some trends: in the absence of Neur, all variants show relatively little internalization, with the exception of DlHA, which is quite well internalized in two of the 10 lineages shown. Upon addition of NeurΔRING, all variants show better internalization, even the lysine/icd2-compromised DlK2R^i2ala^. This supports the role of Neur as an endocytic adaptor, independently of its catalytic domain.

### 3.5. The Dual Role of Neuralized Is Also Evident during Embryonic Neurogenesis

Our results so far have indicated that Neur has a special adaptor function besides its Ub ligase activity and the latter can be bypassed if other sources of ubiquitin are available. Furthermore, ubiquitin can be added onto Delta itself or some other component of the Delta–Neur complex. Our conclusions from the CNS lineages contrast strongly the situation of Notch signaling during wing DV boundary specification, where Delta lysines are important [20]. This is consistent with the fact that *neur* is not expressed in the wing pouch and Delta signaling relies entirely on Mib1. We decided to investigate whether Notch signaling can be executed independently of Delta’s ubiquitylation and Neur’s catalytic function during the selection of embryonic neuroblasts by lateral inhibition. This is another in vivo cell context, where Neur has the predominant role in ligand activation. Classical experiments have shown that *Delta^RevF10^* and *neur^1^* mutations are homozygous embryonic lethal, displaying a “neurogenic” phenotype, namely neural hyperplasia at the expense of ventral epidermis [27]; *Ser* and *mib1* nulls do not cause the same defects [10,38]. Are Delta’s lysines and Neur’s catalytic domain important for this Notch signaling event?

**Figure 8:**
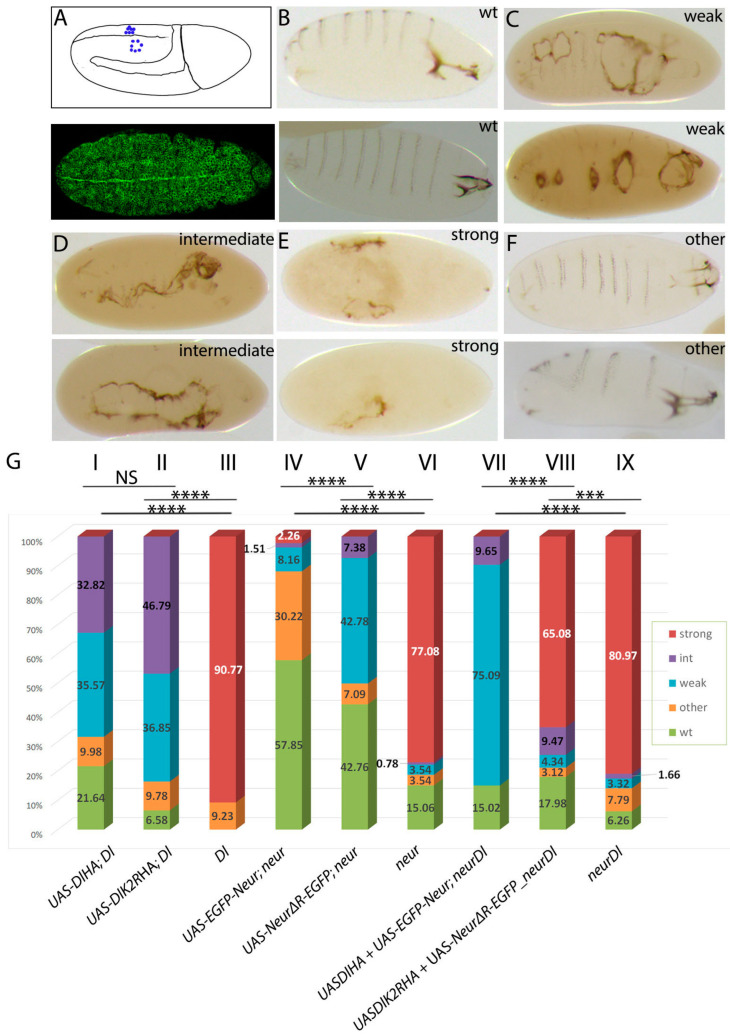
Rescue of the embryo neurogenic phenotype by Delta and Neur variants. (**A**) Top: graphic of a stage 10 embryo indicating individualized NBs (blue dots) in the left side and clustered NBs in the right side, characteristic of the absence of lateral inhibition in Notch pathway mutations. Bottom: da.G32-Gal4; UAS-neurΔRING-EGFP: uniform expression of the transgene (GFP in green) is shown in this superficial ectodermal section of a stage 10 embryo. (**B**–**F**) Cuticle preparations of dead embryos that show increasing lack of epidermis. Wild-type embryos (two examples shown in (**B**)) have normal cephalic structures and denticle belts on their ventral side. Denticle belts are fewer and disorganized in the two examples shown in (**C**) and small holes are present at the ventral side, which arise from a lack of epidermal cells due to mis-specification to neuroblasts. In (**D**), the gaps have fused to each other and the entire ventral epidermis is absent. Finally, in (**E**), almost the entire embryonic ectoderm has transformed to neural tissue, leaving only a tiny fragment of cuticle. We observe also defective embryos with no epidermal holes, like the two presented in (**F**), which show a twisted body plan. We classify these as “other”. In (**A**–**F**), all embryos are shown anterior to the right. (**G**) Percentages of each phenotypic category per genotype. Abbreviations: *Dl* = Delta^RevF10^ mutation, *neur* = *neur^1^* mutation, *neur Dl* = *neur^1^ Delta^RevF10^* mutations. All UAS transgenes are expressed under the control of da.G32-Gal4. Chi-square comparisons showed significant differences (*p* < 0.01) for all experimental samples compared to the relevant control (I, II with III; IV, V with VI; and VII, VIII with IX). Other comparisons are shown above the histogram. NS: no significance; ***: 0.0001 < *p* < 0.001; ****: *p* < 0.0001 Absolute numbers of embryos scored per category, percentages, and all comparisons with their respective *p*-values and χ^2^ are presented analytically in Table S2.

To address this, we expressed either DlK2R-HA or NeurΔRING-EGFP by the ubiquitous driver daughterless-Gal4 (da.G32-Gal4) in *Dl^RevF10^* or *neur^1^* mutant embryos, respectively. We examined two parameters: (a) the number of hatched embryos, to monitor rescue to survival, if any, and (b) the severity of the neurogenic phenotype, based on cuticle preparations. Severity was classified into four different categories, namely wild type, weak, intermediate, and strong, reflecting the increasing loss of the ventral epidermis [39] (Figure 8B–E). We also applied a category named “other”; this corresponded to embryos that possessed ventral epidermis, but showed other anatomical defects, not related to the monitored phenotype (Figure 8F). Regarding the hatching rate, none of the genotypes tested showed rescue to survival, since the observed dead embryos were close to the expected Mendelian ratio of 25%, which corresponds to the homozygous mutant progeny. We also confirmed that the da.G32-Gal4-driven expression of all transgenes (or combinations) studied did not produce embryonic lethality.

Although none of our transgenes rescued embryos to hatching, we observed significant improvements in the neurogenic phenotype (Figure 8B). Both Delta variants, DlHA and DlK2RHA, greatly improved the Delta null phenotype to an intermediate/weak phenotype. In the absence of transgenes, the Delta null phenotype is mostly strong neurogenic. Upon UAS-Dl transgene expression, we even recovered some fully rescued, wt-looking embryos, which, however, did not hatch. The wt-looking embryos were more abundant in the case of DlHA than in DlK2RHA (Figure 8B), suggesting the somewhat compromised activity of lysine-less Delta.

We saw analogous behavior in the substitution experiments with Neur variants. The majority of *neur* mutant embryos are strong neurogenic. This category was eliminated upon Neur variant expression by da.G32-Gal4, whether UAS–neur included the catalytic RING domain or not (Figure 8B). Comparing the two transgenes, the ΔRING version produced a significantly higher percentage of weak neurogenic embryos, which were very rare in the case of the wt EGFPneur variant, which mostly gave wt-looking unhatched embryos. Therefore, the RING domain improves Neur activity during NB specification without being absolutely necessary.

From the above experiments we conclude that lateral inhibition during the selection of embryonic neuroblasts is only mildly affected when we abolish Delta’s ubiquitylation or Neur’s catalytic activity. What would happen if we were to abolish both simultaneously? For this, we co-expressed DlK2R-HA with NeurΔRING-EGFP in *neur^1^Dl^RevF10^* embryos, with the co-expression of their wt counterparts as a control. In these double substitution experiments, the Ub-compromised combination (DlK2RHA+NeurΔRING-EGFP) only modestly reduced the incidence of strongly neurogenic embryos, whereas the Ub-competent combination (Dl-HA+EGFP-neur) eliminated the strong category and instead gave mostly weak phenotypes (Figure 8B). Therefore, in this context, Dl–Neur ubiquitylation seems more important than in the larval CNS. Nevertheless, the Ub-compromised Delta–Neur combination does exhibit detectable activity above the no-transgene condition, suggesting that the adaptor function of Neur is sufficient for a low level of Dl activity even in this context, as in larval GMC.

## 4. Discussion

**Figure 9:**
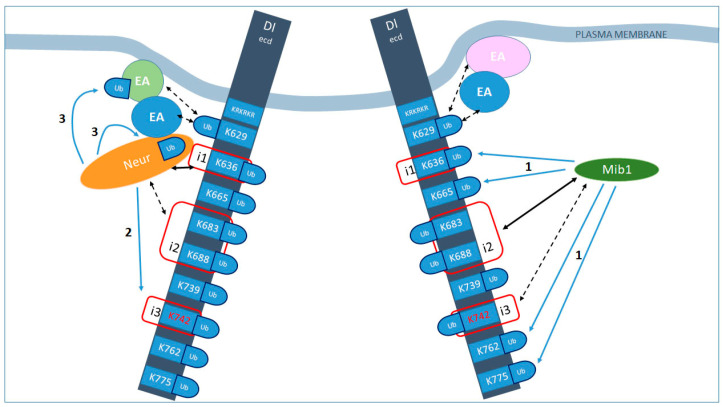
Distinct modes of Delta activation by Neur vs. Mib1. Summary of our working hypothesis of how Neur and Mib1 promote Delta endocytosis and activity. Black arrows indicate interactions, strong (solid) or weak (dashed). Blue arrows indicate ubiquitylations. i1, 2, 3 (red squares) indicate the icd1, 2, 3 motifs of the intracellular domain of Delta. The much larger extracellular domain is not drawn to scale. All lysines of the icd are shown with their residue numbers; all of them are putative Ub acceptors. KRKRKR is the sequence of residues 620–625, which constitute the stop-transfer sequence and could also serve as Ub acceptors. Mib1 interacts with icd2 and 3 [10,18] and can ubiquitylate many Delta lysines (blue arrows labeled 1, exact target lysines have not been mapped). Moreover, this ubiquitylation is needed to promote signaling. Putative endocytic adaptors (EA) are shown that recognize Ub-modified Delta to promote its inclusion in endocytic pits. Neur interacts strongly with icd1 [9] and weakly with icd2 (this work) and ubiquitylates Delta preferentially on K742 (blue arrows labeled 2; [9]), but can also ubiquitylate another substrate (blue arrows labeled 3; this work). It is also an endocytic adaptor itself, shown in this schematic by its direct contact both with the Delta cargo and with other EAs. The presence of Ub molecules, although not absolutely necessary, enhances the activity of Delta, perhaps by strengthening the formation of such adaptor–cargo complexes and promoting the efficient endocytosis of Delta.

As in several other cases of asymmetric cell division, neuronal siblings produced upon GMC division signal to each other via Notch. We dissected the requirements for Delta and Neur domains during this instance of inter-sibling Notch signaling, using the expression of Hey as a reporter of the Notch-ON state. The major findings reported herein are summarized in Figure 9 and are the following: (1) Neur most likely acts in a complex with Delta to enable its signaling; this Delta–Neur complex forms using two Delta motifs, icd1 (strong) and icd2 (weak); (2) robust Delta–Neur signaling needs ubiquitin in addition to Neur; (3) there appears to be a certain degree of liberty on the precise attachment site(s) of ubiquitin on the Delta–Neur complex and on the enzyme that catalyzes this attachment; namely, ubiquitin can enhance Delta–Neur activity even if it is not catalyzed by Neur itself; furthermore, ubiquitin does not necessarily have to be added on the Delta lysines; (4) Delta–Neur acts in a dynamin-dependent but Epsin-independent manner.

The need for force generation by the ligand to activate Notch is supported by many lines of evidence (reviewed in Seib et al. [24] and Sprinzak et al. [40]). This is usually achieved via ligand endocytosis. Taken together with previous results [20,22,30], our work suggests that Mib1-mediated Delta signaling relies on the Delta lysines, whereas Neur–Delta does not. The dispensability of Delta lysines is in agreement with recent results showing that a DlK2R variant knocked into the endogenous locus by CRISPR/Cas9 completely rescues the lack of endogenous Delta (normally lethal) and yields homozygous viable adults with only small defects in wings, legs, and oogenesis [41].

Besides the catalytic RING domain, the Mib1 and Neur proteins are structurally very different [17,42]. We propose that Neur associates stably with Delta (and actively participates in its endocytosis), whereas Mib1 binds transiently to catalyze Delta ubiquitylation. We base this hypothesis on three key observations: (1) the fact that a ΔRING version of Mib1 is always dominant negative [12], whereas NeurΔRING often enables Delta activity (this work and references [8,12]); (2) the defects seen in CRISPR knock-in DlK2R flies are all in Mib1-dependent biological contexts [41]; (3) Neur often colocalizes with Delta in intracellular puncta in a Delta-dependent manner, suggesting that the two proteins associate and co-traffic in the endosomal pathway [9,12,18]—this is not observed with Mib1 [13]. This Delta–Neur endocytic complex is likely to be the active form of Delta and signals weakly even without any ubiquitin (the DlK2R^i2ala^+NeurΔRING combination, Figure 6). However, to reach full activity, the Delta–Neur complex needs ubiquitylation: this is normally provided by the Neur catalytic domain, which seems to ubiquitylate both Delta [9] and some other member of the complex (perhaps Neur itself [18]). This incremental improvement in signaling by ubiquitin is consistent with a recent preprint that shows evidence that Ub contributes to the multivalent interactions of early endocytic adaptors with their cargo and enhances the efficiency of coated pit formation [43]. Ubiquitin may increase Delta–Neur activity either by strengthening the endocytic pulling force or by enabling Delta–Neur recycling to dislodge the ligand from cis complexes with Notch, which are known to inhibit signaling [24]. Whatever the mechanism of ubiquitin enhancement might be, if Neur is catalytically inactive, ubiquitin can be provided by Mib1 on Delta lysines, achieving an equivalent enhancement in Delta–Neur activity. Given the involvement of ubiquitin, it is surprising that Epsin is not needed in the context of GMC inter-sibling signaling, even though it is needed in most other cases of DSL–Notch events, whether Mib1- or Neur-dependent [10,22,37]. Another instance where Epsin seems to be dispensable is the inter-sibling signaling between pIIa and pIIb, the progenies of sensory organ precursor division [30]. It remains to be discovered which ubiquitin-binding endocytic adaptors mediate these two asymmetric cell division events.

Besides the mechanistic details of co-trafficking and ubiquitin attachment, Delta–Neur signaling seems to be stronger than Delta–Mib1. This has been noted before in the wing epithelium, where Neur is not normally expressed but has been studied in artificial settings [12,22]—ectopic Neur enables Delta signaling far from the DV boundary, where Delta alone (acting via Mib1) would have been unable to induce Wg or Cut expression. It has also been observed in the present work, where Neur-independent but endocytosis-competent ligands (namely Dl-LDL+ and Dl^i1ala^) are weakly active or inactive, despite the fact that Mib1 is present. It is possible that a weak Delta pulling force is insufficient in the CNS setting, but is sufficient to allosterically deform Notch in the wing epithelium, where there is appreciable apical tension among the cells [44]. In the more parenchymal neural tissue, the opposing cell poses less resistance, so a stronger pulling force is expected to be required to produce sufficient ligand displacement to deform the Notch receptor. It should be noted that Delta-endocytosis-independent Notch activation has been reported in tissue culture cells where a Delta ligand has been immobilized to the stiff support of the culture dish [45]. It is also noteworthy that Neur has evolved another type of activity, independently of Delta–Notch, which is to promote epithelial loosening via degradation of the Crumbs–Stardust complex [46,47]. Perhaps events of epithelial to mesenchymal transition triggered by Neur have co-opted Delta expression to accompany this tissue remodeling with the emission of a Delta–Notch signal (e.g., Contreras et al. [48]). One is left wondering why Neurl1 and 2, the mammalian homologues of Neur, have not been implicated in instances of Notch signaling, especially those associated with tissue remodeling. A way forward would be to study the Neur–Delta–Notch axis more thoroughly in vertebrate non-mammals, where there is already evidence of a mechanism that could be similar to that in *Drosophila* [18].

## Figures and Tables

**Table 1:** Site-directed mutagenesis primers.

Mutation	Primer Name	Primer Sequence
NEQNAV to AAAAAA (Dl^i1ala^)	Dl_sdm_NxxN2A_fwd	G GCC AGG AAG CAG GCC GCA GCG GCT GCG GCG GCC ACA ATG CAT C
	Dl_sdm_NxxN2A_rev	G ATG CAT TGT GGC CGC CGC AGC CGC TGC GGC CTG CTT CCT GGC C
IRNTWDR to AAAAAAA (DlK2R^i2ala^)	DlK2R_sdm_NBox2A_fwd	GGC GGC AAC CCG AAT ATC GCC GCT GCC GCC GCG GCC GCA TCG GTC AAC AAC ATT TGT GCC
	DlK2R_sdm_NBox2A_rev	GGC ACA AAT GTT GTT GAC CGA TGC GGC CGC GGC GGC AGC GGC GAT ATT CGG GTT GCC GCC

## Data Availability

The raw imaging data presented in this study are available on request from the corresponding author.

## Supplementary Materials

The following supporting information can be downloaded at: https://www.mdpi.com/article/10.3390/cells12242833/s1, Figure S1: Expression patterns of DlGFSTF, SerGFSTF, and NeurGFSTF lines in the CNS of third-instar larvae; Figure S2: The deletion mutant Dli1 shows similar levels of activity with the point mutant Dl^i1ala^; Figure S3: NeurΔRING enhances endocytosis of Dl, Dl^i2ala^, DlK2R, and DlK2R^i2ala^. Table S1: GMC-Asymmetric Division Assay Statistics; Table S2: Embryo-Lateral Inhibition Assay Statistics.
