# Supplementary figures and images for: Separable Roles for Neur and Ubiquitin in Delta Signalling in the Drosophila CNS Lineages
Source: Cells. 2023 Dec 14;12(24):2833. doi: 10.3390/cells12242833 (PMC10741450; doi:10.3390/cells12242833)

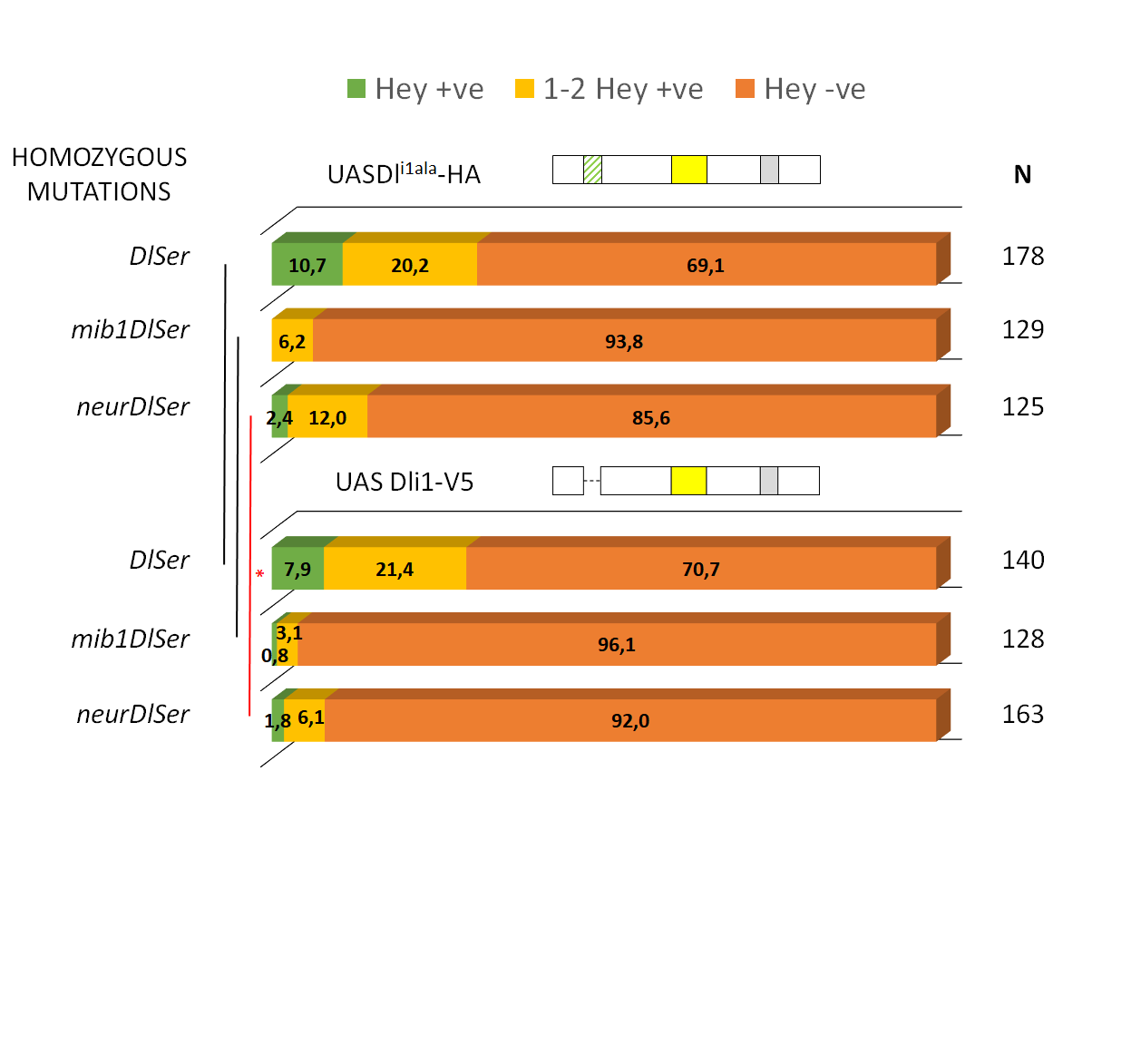

Supplement: Supplementary file 1 [file cells-12-02833-s001.zip › Supplementary Figure S2_.tif]

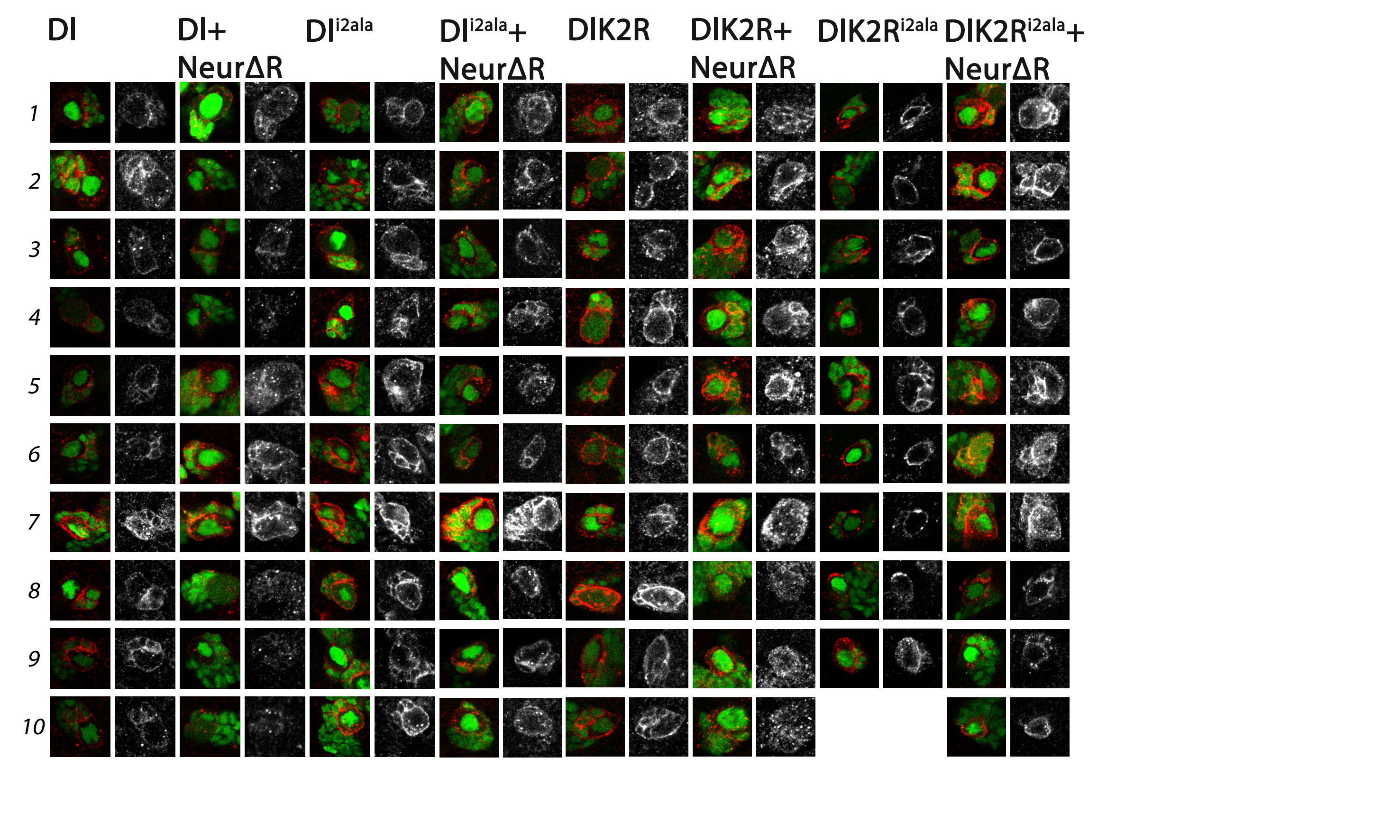

Supplement: Supplementary file 1 [file cells-12-02833-s001.zip › Supplementary Figure S3_.tif]
